# Spontaneous Fusion of L5/S1 Spondylolisthesis in an Elderly Female: A Case Report

**DOI:** 10.7759/cureus.32863

**Published:** 2022-12-23

**Authors:** Elham A Alghamdi, Muhammad R Amin Khan, Abdullah Hamad, Ali A Alzahrani

**Affiliations:** 1 Orthopedic Surgery, King Saud Medical City, Riyadh, SAU; 2 General Practice, King Saud Medical City, Riyadh, SAU

**Keywords:** conservative management, lumbosacral, fusion, spontaneous, spondyloptosis

## Abstract

We report a case of a 65-year-old female presenting with an Anterolisthesis grade I, L5-S1. With a history of lower back pain that started two years ago with weak big toe extension. CT scan revealed that There is anterolisthesis grade I, L5-S1. No pars defect was seen, and degenerative changes in the bilateral facet joint L5-S1, with narrow joint space & sclerosis. The patient underwent conservative management to strengthen and stretch her back muscles for three months and had spontaneous fusion develop at an unstable level with relief of symptoms after nonoperative treatment.

## Introduction

A non-traumatic progressive disruption of two adjacent vertebrae is classified according to the Meyerding classification caused by the slippage of the spinal column. In contrast, in traumatic cases, a disruption of the posterior ligament-osseous structures occurs [1]. Based on a literature review determined that 85%-95% of the cases of spondylolysis and spondylolisthesis develop at the level of the L5 vertebrae and 5%-15% at the level of the L4 vertebrae, with no correlation detected between spinal level and grade of spondylolisthesis [2,3]. An additional prospective study found that males were twofold affected than females [4].

## Case presentation

We report the case of a 65-year-old Saudi female known to have diabetes mellitus and hypertension, which were controlled with regular medications. Presented with generalized body pain for four years, associated with back pain for two years that is worsening and increasing gradually over time. She described the back pain as continuous, sharp with heaviness in nature, moderate in intensity (5/10), relieved by rest, and aggravated by walking and bending. The patient also experienced pain in the gluteal region, mild thigh pain, and weakness in the right big toe. As part of the examination, a general physical examination was normal. Upper limbs had no abnormalities on spinal examination. The Modified Schober test [5] was negative. The muscle power scale in both lower limbs was 5/5, apart from L5 demonstrating weak Rt big toe extension with a power rating of 2/5. In the patient's sensations examination, all dermatomal levels were intact, no peripheral neuropathy, and the patient was able to control her bowel and bladder sphincters normally. The patient walks on heels freely, stands on her toes with assistance, and squats with difficulty. Further investigations for generalized body pain such as vitamin deficiency and osteopenia/porosis have been excluded.

**Figure 1:**
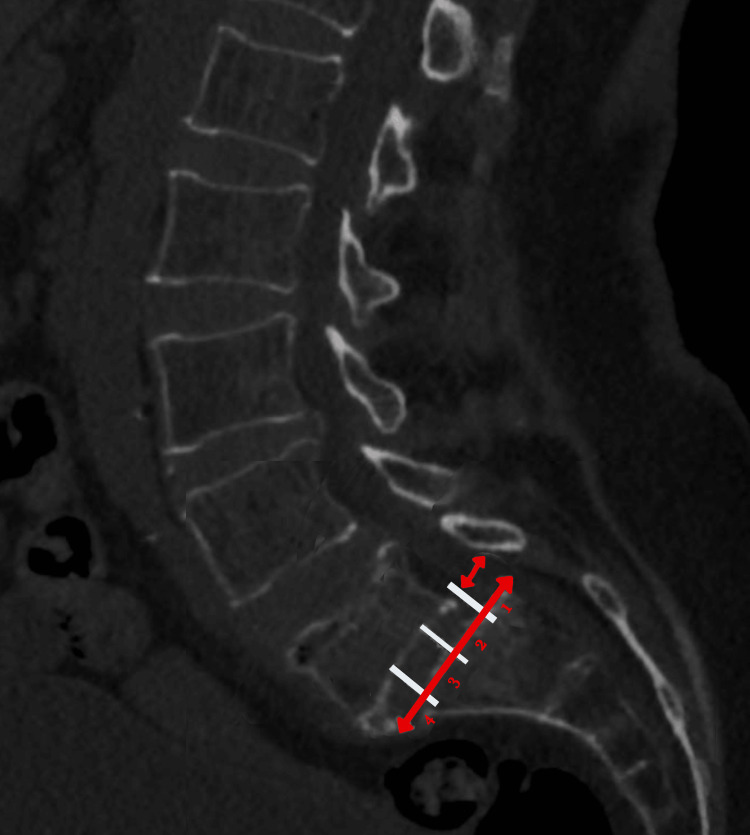
CT anterolisthesis grade I at the level of L5-S1

**Figure 2:**
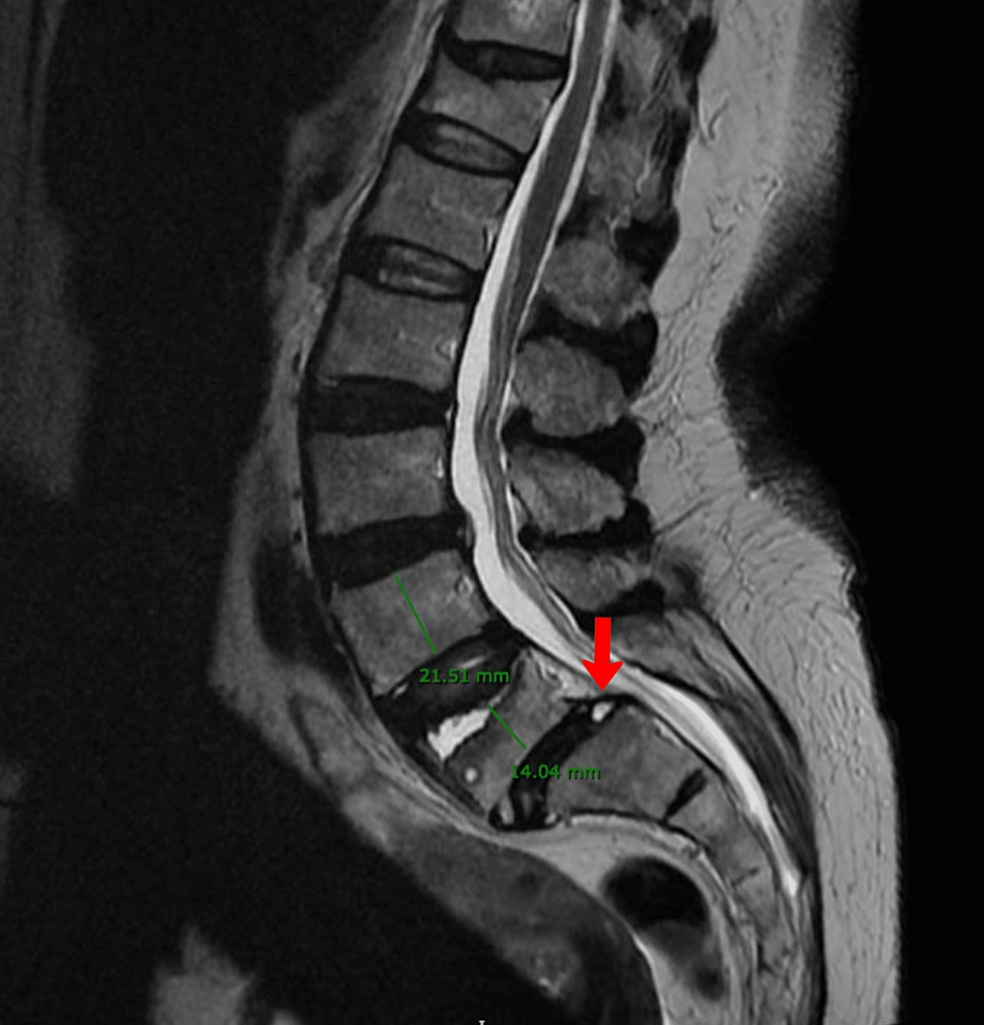
MRI with preserved lordotic curvature with first-degree spondylolisthesis over S1 and decreased height of L5

A CT scan identified anterolisthesis grade I at the level of L5-S1. No pars defect has been found, but degenerative changes have been observed in bilateral facet joints of L5-S1, resulting in narrow joint spaces and sclerosis. There is bilateral osteoarthropathy of the other visible vertebrae and a vacuum phenomenon at L4-5. A relative exaggeration of lumbar lordosis is seen with diffuse posterior disc bulge at L4-L5 and L5-S1, with bilateral nerve exit foramen compromise (Figure 1). A lumbar MRI (Figure 2) demonstrated preserved lordotic curvature with 1st-degree spondylolisthesis over S1 and decreased height of L5. Moreover, the posterior disc bulge between L5 and S1 indents the ventral aspect of the thecal sac and infringes on the lower portion of both exit neural foramina. The cauda equina and conus medullaris were in normal condition, and there were no abnormalities of the soft tissues in the paravertebral region.

The patient was advised to go for rehab stretching and strengthening back muscles with soft lumbar support. Follow-up after three months of conservative management helped to improve the patient clinically regarding the pain; on the other hand, she had taken analgesics as home medications, with regular follow-up annually with imaging for observing further spontaneous fusions [6].

## Discussion

Spondylolisthesis is described as a translation of a vertebra with respect to the vertebra below without any modification or notable lesion to the pars interarticularis. The combination of abnormal weight distribution and soft tissue laxity and instability over a prolonged period leads to excessive joint play and buckling of the posterior annular fibers of the intervertebral disc. In addition to causing stresses and strain to the neural tissues of the spine, abnormal spinal alignment and posture also cause more strain on the spinal cord's vascular supply. Consequently, this can affect the body's motor, sensory, and autonomic nervous systems [7]. A large retrospective study done in 2014 reported that 20.9% of spontaneous fusions have been found in radiological studies during follow-up, with no specific relationship between the level or grade of spondylolisthesis [4]. Generally, patients who suffer from grade I or II spondylolisthesis should be managed conservatively if their daily activities are not impaired. Excellent results can be achieved When very early or early spondylolysis is treated with conservative management methods such as bracing, activity restriction, and Physiotherapy. Based on a literature review by Nabil et al., surgical management of symptomatic patients achieves a similar quality of life to those patients with minimal symptoms treated conservatively, and surgical treatment delays did not negatively affect patient outcomes [2]. This case report presented an old-aged woman with isthmic spondylolisthesis grade I, manifested with isolated neurological weakness but was not affecting her daily live activity, so chose to go for conservative treatment, observed during three months of regular follow-up. It is possible to achieve correct spinal alignment without surgical intervention. However, there were no reported cases in Saudi Arabia of the spontaneous fusion of spondylolisthesis with the improvement of the neurological deficit under conservative management [6].

## Conclusions

In order to treat spinal deformities that present with weakness effectively, surgeons should not ignore conservative treatment without first starting with the basics. In our case, conservative treatment consisted of reducing the mechanical stress over the lumbar spine and improving muscle strength to alleviate pain and disability.
